# State Examination for License

**Published:** 1893-11

**Authors:** 


### BUFFALO MEDICAL AND SURGICAL JOURNAL

A MONTHLY REVIEW OF MEDICINE AND SURGERY.

*EDITORS:*

THOMAS LOTHROP, M. D. - - WM. WARREN POTTER, M. D.

All communications, whether of a literary or business character, should be addressed to the managing editor: 284 FRANKLIN STREET, BUFFALO, N. Y.

VOL. XXXIII. NOVEMBER, 1893. No. 4.

### STATE EXAMINATION FOR LICENSE.

WE have before us a copy of the law passed by the Pennsylvania legislature, regulating the practice of medicine in that State. The act is largely modeled after our own State law, but contains some features which we fear may prove a stumbling-block, because of the non-existence of a board of educational control, such as is our state board of regents. A medical council, composed largely of state officers, will be the governing body, who are to supervise the examinations, both academic and professional. And just here is where the trouble is likely to arise. If the work of this council is to be rendered effective, the standing of every educational institution in this country, and the regulations governing foreign schools of learning, must become a part of its office files. The appropriation of moneys for all the purposes of the law is but one thousand dollars per year, which sum includes the salary of the secretary and treasurer. The law is to go into effect March 1, 1894, and if those interested are wise, they will ask the regents at Albany to place at the disposal of the Pennsylvania Medical Council the records, accumulated during the past 100 years, which show the institutions whose certificates indicate the completion of a "competent common school education," and also the rating of the many medical colleges in as well as out of the country.

As in this state, there are to be three boards of examiners, representing the three incorporated systems of medicine. In almost every other particular the law follows closely the lines adopted in our state, and many pitfalls will be avoided if the newly-appointed examiners were to make a practical inspection of the workings of the law in New York. If the same harmony which marks the operation of the law in this state, obtains in

Pennsylvania, we predict great and lasting results as the outcome of this latest step in the advancement of medical science.

Two years have rolled by since the law empowering the state *alone* to grant license for medical practice, has been in operation in New York, and the promises of the advocates of the measure have been more than fulfilled. The situation, fumed at by charlatan, fretted over by frightened student, and fought against by some college professors, has now become a matter of fact, and we hear of no one other than the barnacle that fattens in the absence of restraining influences, who is willing to return to the old order of things, which existed for so many years. It is safe to say that in less than ten years the distinctions, now barely understood, or for the most part very much misunderstood, by the laity, which in medicine, as in all science, recognizes only worth, and relegates bluster and charlatanry to the rear, will be an open book, so that he who runs may read. The advertising quack will have his place as does the shyster lawyer, because all *physicians* must needs by that time be on a parity, the foundation of which will be knowledge, vouched for by the state in the form of a license.

Most of the exemptions having ceased, the last academic year was a busy one for the New York State medical examiners. There were 327 candidates for license, showing that the estimate of an annual influx of 400 physicians, rather than from 600 to 700 as of old, is very near the mark. It is more than probable that the current year will demonstrate this still more clearly. Of this number, 267 succeeded in meeting all requirements and passing the medical examinations, and are now registered as practitioners of medicine by virtue of a state license. Two hundred and forty-four of these are state board men, seventeen are homeopaths, and six are eclectics.

Many persons suppose that the position of state medical examiner is a sinecure, and that from the fees collected a revenue of no mean proportions is returned to the individuals constituting the boards. It will be interesting, therefore, for such to study the following figures :

| | |
|---|---|
| Gross income since September 1, 1891 ............ | $10,005.00 |
| Gross expenses of enforcing the law and conducting the examinations, disbursed by the regents.... | 7,835.40 |
| Balance................................ | $2,169.60 |

Divided among the examiners *pro rata*, according to the number of candidates examined, this has yielded to examiners representing the Medical Society of the State of New York, $246.21, or $123.10 each, per annum; to those representing Homeopathic State Medical Society, $163.17; and to those representing Eclectic State Medical Society, $54.40.

The syllabus now in course of preparation promises to be a work of large proportions, and likely to be a standard for study in most, if not all, of the colleges in the country. The demand for the last one was very great, but its compilation was hurried and calculated more for the use of out-of-the-state practitioners of long standing than for the recent graduates; hence the text necessarily could not be "up to date." The work now in press will be a scientific model for the systematic study of medicine, as well as a guide for those who contemplate taking the state licensing examinations.

Some of the effects of the establishment of state boards of medical examiners have been improved methods of teaching, prolongation of terms of study, and the establishment of new chairs of instruction. While in New York State the entire subject of medicine is divided into seven main topics, the questions asked under these topics are so sub-divided as to comprehend almost every sub-head in medicine. Realizing the importance of having their graduates well equipped for passing these tests, the means above enumerated have been adopted for the benefit of college and profession. In consequence, teachers on hygiene, medical jurisprudence, and other sub-divisions of medicine, exist now in fact as well as in name in most of our colleges, a thorough drill and review teaching is a feature of the curriculum, and some of the lecture courses extend over a period of eight months.

Under the direction of the Committee on Legislation of the Medical Society of the State of New York, an auxiliary committee has been at work looking out for the enforcement of the law governing medical registration in every county in the state. They have done such excellent work, that today every registration recorded since the new law went into effect, is known to the society, and where correction of illegal registration has not already been made, the penalty of the law will be enforced. In addition, county clerks are now conversant with the conditions regulating registration, and it is more than certain that no new name will be added to the records unless the document on which registration is demanded, bears the seal of the regents.

Are you a graduate of an out-of-the-state medical college? Was your diploma indorsed by the faculty of a New York State medical college, or by the regents previous to your registration in this state? If not, you are practising medicine illegally; your evidence as a witness may be brushed aside whenever you attempt to give medical testimony; you cannot collect a claim for professional services; you are liable to prosecution for illegal practice. The remedy lies in making immediate application for validation of your illegal registration. This can be done only on the recommendation of the state board of medical examiners. If this fits your case, write at once to Regents' Office, Albany, N. Y., for proper blanks and a copy of the law,—you will find provision made for you in par. 148, sec. 2, ch. 661, Art. 8, Laws of 1893.

Under the new law it is well to remember that every document presented at a county clerk's office, in order to be registered, must bear the impress of the Regents' seal. This seems on its face like the extreme of red-tape, but such is not the case. The man whose original registration was valid, may have the seal applied to his credentials on application and with only such loss of time as is entailed by the transmission of his documents or diploma to and from Albany. Every reputable practitioner changing his residence from one county to another is willing to take this little trouble to place himself on a safe footing. On the other hand, the quack, if he succeeded at some previous time in being registered illegally by some pliant or ignorant official, now finds his field of operations confined to the one county, as an appeal for the authoritative seal for practice elsewhere, at once exposes the irregularity of his original registration and renders him liable to prosecution.

